# The Position of the Ovaries

**Published:** 1882-07

**Authors:** 


### THE POSITION OF THE OVARIES.

Professor His, quoted by Dr. Dwight (Boston *Medical and Surgical Journal*, March 9, 1882), contributes a new paper to the increasing literature of this vexed question. He holds, in the main, to his theory, that they lie with one side resting against the wall of the true pelvis, and that the long axis is vertical, a view which appears to have gained ground since its publication in 1878. He has since very carefully examined the position of these organs in three suicides aged respectively fifteen, sixteen and twenty years, and finds, as indeed might have been expected, that if the fundus of the uterus is on one side of the median line, the ovary of the opposite side is drawn downward and into an oblique position. It is evident that after this position has once been reached, the weight of the viscera will tend to press the ovary more and more from its normal position. Summing up the results of all his observations, His gives the following as the normal or primary position of the ovary in the young adult female. The organ lies with its side against the wall of the pelvis, the free border being behind and the attached end below. The Fallopian tube is looped over the ovary, rising along the front and falling behind it. The fimbriæ then turn back and spread over the summit of the ovary. It should be remembered that with advancing years and after repeated pregnancies the situation of the ovary must become more and more uncertain.—*Chic. Med. Rev.*

